# Effect of Exercise on Quality of Life in Parkinson's Disease: A Systematic Review and Meta-Analysis

**DOI:** 10.1155/2020/3257623

**Published:** 2020-07-09

**Authors:** Kui Chen, Yan Tan, You Lu, Jiayan Wu, Xueyuan Liu, Yanxin Zhao

**Affiliations:** Department of Neurology, Shanghai Tenth People's Hospital, Tongji University, School of Medicine, Shanghai, China

## Abstract

**Background:**

Exercise has an integral impact on the physical and mental wellbeing of patients with Parkinson's disease (PD), yet no comprehensive and quantitative analysis has been conducted on the effect of exercise on quality of life (QoL) in these patients. This study aimed to evaluate the effect of exercise on overall QoL and different domains of QoL in people with PD, as well as investigating the influence of factors such as the exercise type and intervention period.

**Methods:**

Databases, such as PubMed, Embase, and Cochrane Central Register of Controlled Trials, were searched since inception to August 14, 2018 to identify randomized controlled trials that compare the effect of exercise versus no intervention on QoL in PD patients. Following the subgroup analysis, heterogeneity was further explored. The quality of eligible studies was assessed according to PRISMA guidelines.

**Results:**

20 studies were included with 1,143 participants in total. A meta-analysis showed a significant improvement in QoL after exercise intervention in PD patients (SMD = −0.24, 95% CI = −0.36 to −0.12, *P* < 0.001). A subgroup analysis of exercise types revealed significant QoL improvement with aerobic exercise, martial arts, and dance, but not anaerobic exercise and combined exercise. Interventions lasting 12 weeks or longer improved QoL significantly.

**Conclusions:**

Exercise interventions, especially aerobic exercise, dance, and Tai Chi, significantly improve QoL in PD patients. At least 12 weeks of exercise is needed to bring about significant benefits.

## 1. Introduction

Parkinson's disease (PD) is a neurodegenerative disease jeopardizing mobility, mental health, and social interaction. In recent decades, interest in quality of life (QoL) of PD patients has grown, with clinical focus shifting from how well patients are moving to how well they are living. To this end, scales have been developed to assess overall as well as detailed aspects of QoL. The 39-item Parkinson's Disease Questionnaire (PDQ-39) is the most widely used QoL scale specific to PD patients [1, 2]. The basis of PD therapy is pharmacological intervention [3]. Physical exercise represents a complementary treatment option, yet previous studies have failed to demonstrate its uniform benefit on mobility [4, 5]. However, because exercise is a multidimensional activity with combined effects on both physical and mental wellbeing, it would be more reasonable to assess the impact of exercise by evaluating QoL changes. Indeed, several researchers have shown that exercise plays an important role in relieving suffering and improving QoL as a supplement to drug therapy [6, 7].

Evidence from previous study regarding the influence of exercise on QoL varies considerably. De Silva et al. conducted a systematic review of randomized controlled trials (RCTs), reaching the qualitative conclusion that physical exercise-based rehabilitation programs promoted the QoL of PD patients [8]. However, this study lacks the quantitative analysis. In addition, there is no analysis of how different exercise types contribute to QoL and how exercise benefits different domains. The present review consists of a quantitative synthesis of the overall effect of exercise on QoL in PD patients, and it identifies factors that accounted for the variation. In addition, we explored the influence of exercise on different QoL domains.

## 2. Methods

This systematic review and meta-analysis complied with the guidelines of Preferred Reporting Items for Systematic Reviews and Meta-Analyses (PRISMA) [9]. The review protocol has been registered at the international prospective register of systematic review (PROSPERO, CRD42019138639).

### 2.1. Eligibility Criteria

We aimed to evaluate the effect of exercise on the QoL of PD patients. In order to define the remit of our meta-analysis, the participants, interventions, comparisons, outcomes, and study design (PICOS) framework was applied as follows. (*P*) Participants were patients diagnosed with PD, over the age of 18, and able to take exercise intervention. (*I*) Exercise interventions were defined as structured and planned systemic physical activities at an effort above activities of daily living to improve balance, functionality, and mobility and were divided into five categories: aerobic exercise, anaerobic exercise, dance, martial arts, and the combined exercise of anaerobic and aerobic training. (*C*) The patients in control groups maintained their usual lifestyle and current levels of the physical activity, took no extra exercise or training, and could be provided with nonexercise health education or advice. (*O*) Changes in QoL from baseline to the first follow-up point after intervention were reported using QoL scales such as PDQ-39 [1], Parkinson's disease quality of life questionnaire (PDQL) [10], EuroQol (EQ-5D) [11], the short version of the Sickness Impact Profile (SIP-68) [12], the 12-Item Short Form Health Survey (SF-12) [13], and the WHO Quality of Life questionnaire (WHOQOL) [14]. (S) The study design was an RCT including the first phase of a crossover trial.

### 2.2. Information Source and Search Strategy

We identified relevant studies by searching general medical and science databases (PubMed, Embase, and Web of Science), trial registries (Cochrane Central Register of Controlled Trials, Clinical Trials, and ISRCTN registry), dissertation databases (ProQuest, EThOS, DART-Europe, and NDLTD), physiotherapy evidence database (PEDro), and conference and grey literature databases (Open Grey, GreyNet, Grey Literature Report, and Conference proceedings citation index) from database inception until August 14, 2018 without language limitations. The search strategy was based on the following words, phrases, and MeSH terms: “Parkinson” or “Parkinson's disease;” “exercise,” “physical activity,” “sport” or “dance;” “quality of life” or “life quality;” and “clinical trial” or “random”. A detailed search strategy is described in Supplementary Appendix 1, using PubMed as an example. Finally, manual searches of the reference lists of eligible articles, systematic reviews, and meta-analyses were completed.

### 2.3. Study Selection

Pertinent studies were assessed by two independent reviewers in three steps. First, duplicate studies were removed. Second, through the evaluation of the titles and abstracts, unqualified studies were removed based on the eligibility criteria. Third, the full texts of the remaining studies were retrieved for more detailed evaluation according to the same eligibility criteria. During the process, any disagreement between reviewers over the eligibility of particular studies was resolved through discussion with a third reviewer.

### 2.4. Data Extraction and Quality Assessment

Information regarding study design and setting, study population (number of participants, age, sex, and other characteristics at baseline), exercise intervention (type of exercise, exercise intensity, and total duration), control conditions, measurement of QoL (instrument used, time of assessment, and change in QoL), and information for assessment of the risk of bias (ROB) were extracted from the full text of the included studies using a piloted table by two reviewers independently. The risk of bias of included studies was assessed by two reviewers using the Cochrane Collaboration's tool [15]. Discrepancies were identified and resolved through discussion (with a third reviewer where required).

### 2.5. Data Synthesis and Analysis

Measurements of QoL were extracted from included studies. Considering that instruments evaluating QoL were different among studies, the standardized mean difference (SMD) was applied. In light of the potential heterogeneity of participants, intervention types, and instruments for outcome measurement, a random effects model was more appropriate for the outcome analysis rather than a fixed model. Heterogeneity was quantified with Cochran's *Q* test and *I*^2^ statistics. The sensitivity analysis was conducted to examine the impact of an individual study on the overall effect. Publication bias was qualitatively assessed by a funnel plot and quantitatively analyzed by Egger's and Begg's tests. Subgroup analyses were conducted according to predefined variables (study populations, exercise type, intervention duration, and rating scales of QoL). If necessary, a meta-regression analysis would be performed. Effect size estimates with two-sided *P* values <0.05 were considered statistically significant. Analyses were performed using Stata, version 15.0 (StataCorp, College Station, Texas, USA).

## 3. Results

### 3.1. Study Selection

**Figure 1:**
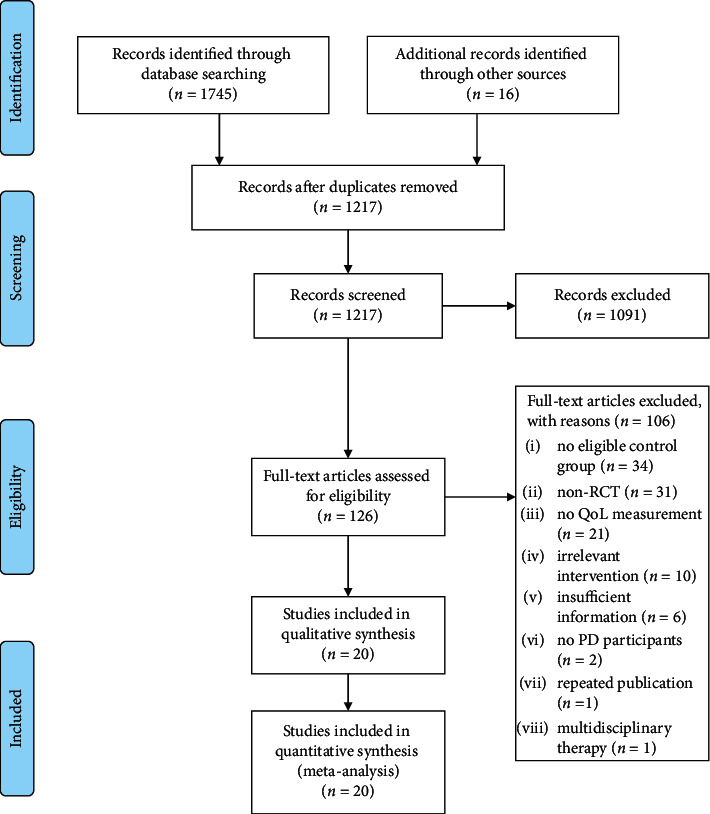
Flowchart for the systematic review and meta-analysis.

The initial search of all databases retrieved 1,761 studies. The studies were selected as shown in the flowchart (Figure 1). 20 studies that met the inclusion criteria were evaluated in this meta-analysis [6, 7, 16–33].

### 3.2. Description of the Included Studies

Among included studies, there were four multiarm trials with two or more eligible experimental groups [18, 22, 25, 30]. In two of these four trials, the exercise interventions were considered similar (resistance training and resistance training with instability [30]; tango and waltz/foxtrot [22]), so the data from the two exercise arms within each trial were integrated to gain one comparison versus no intervention. As a result, 20 studies contributed data to 23 comparisons.

Of the 20 studies, seven were conducted in North America, six in Oceania, five in Europe, one in South America, and one in Asia. The number of participants in the 20 trials ranged from 15 to 211, with 1,143 participants in total. The number of drop-outs before first assessment ranged from 0 to 14. The mean age of participants was 68.0 years. 61.3% of the participants were male. The mean disease duration was 6.7 years, and the mean baseline Unified Parkinson's Disease Rating Scale Part III (UPDRS III) was 24.2. There were three studies conducting anaerobic exercise as intervention. Each of the following exercise interventions: aerobic exercise, mixed exercise, martial arts, and dance, was performed by five studies. The duration of intervention varied from 3 to 25 weeks. QoL was evaluated by PDQ-39, PDQL, EQ-5D, or SIP-68 in these trials. The data at baseline and the first assessment point in each trial were collected. The characteristics of all studies included in this review are summarized in Table 1. Several studies did not provide enough details regarding the characteristics of their samples.

### 3.3. Risk of Bias

The quality of eligible studies and a detailed quality assessment for each included study are shown in Supplementary Appendix 2. Five studies were categorized as having a high risk of other bias. In the trial by Canning et al., the final sample size was 20 patients, which was far fewer than the recruitment target of 140 due to lack of ongoing funding [17]. Participants in the study by Cruise et al. were recruited from PD support groups and so were likely more proactive than the general PD patient population in support seeking and disease management [20]. The study by Vergara-Diaz et al. was a two-arm, waitlist-controlled RCT with a six-month intervention duration, but only the assessment at three months was taken into consideration in this review [7]. In the trial by Hackney and Earhart, patients participating in the Tai chi group may have had slightly more dyskinesia than those in the control group (*P*=0.05) [22]. In the study by McKee and Hackney, there was a statistically significant six-year age difference between groups [24].

### 3.4. Effect of Exercise on QoL in PD

**Figure 2:**
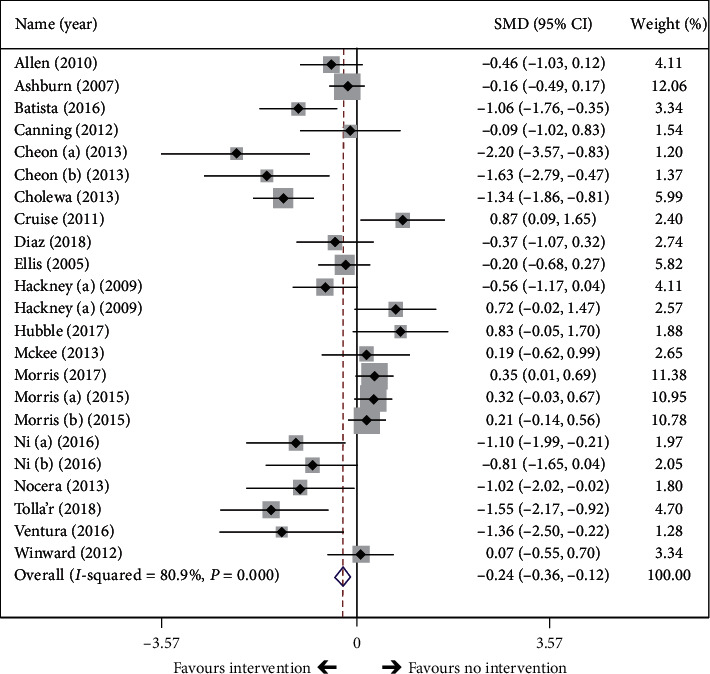
Effect of exercise intervention on overall quality of life in PD patients. The black diamond represents the standardized mean difference (SMD) for each trial with the arms reflecting 95% confidence intervals (CIs). The *x* axis scale indicates the range of 95% CIs of the trials. The size of the shaded box reflects the relative weight of each study. The unfilled diamond represents the overall SMD of the trials, and its width represents the confidence interval.

A random effects meta-analysis showed significantly decreased scores of QoL with exercise compared to no exercise intervention (SMD = −0.24, 95% CI = −0.36 to −0.12, *P* < 0.001, 23 comparisons, Figure 2) and indicated an improvement in QoL in favour of exercise. High heterogeneity was observed in the analysis (*Q* = 115.10, d*f* = 22, *P* < 0.001, *I*^2^ = 80.9%).

**Figure 3:**
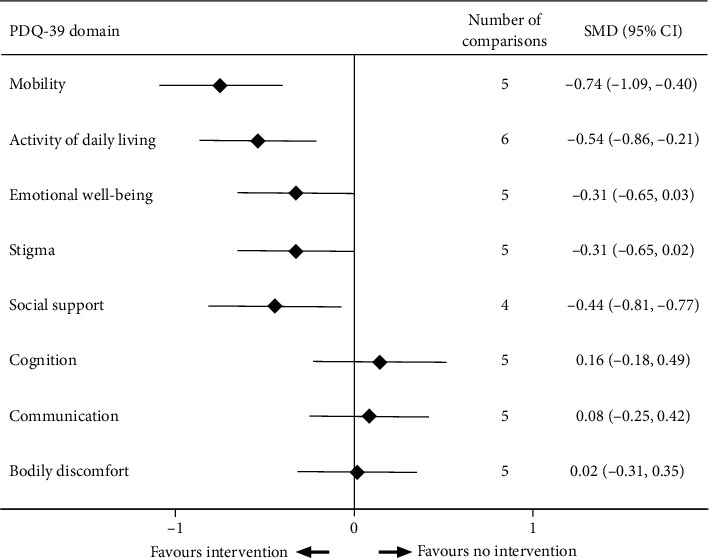
Effect of exercise intervention on eight domains of quality of life in PD patients.

Aside from total scores of QoL, five trials reported detailed scores of domains in PDQ-39 [22, 27–29, 32]. Since one of these studies was a multiarm trial [22], data from six comparisons were obtained. One trial only showed the results of the activities of daily living (ADL) domain [32]. In addition, one trial showed the mean change of scores on the social support domain in the control group as 0.0 (SD = 0.0) [29], and this was excluded from the analysis. Thus, the effects of exercise on six QoL domains were calculated through five comparisons, except for the ADL domain (six comparisons) and social support domain (four comparisons) (Figure 3).

Results of analysis by domain indicated that exercise was associated with significant improvements in the domains of mobility, ADL, and social support. The pooled effect of exercise was −0.74 on the domain of mobility (*P* < 0.001, *I*^2^ = 41.8%, 5 comparisons); −0.59 on the domain of ADL (*P*=0.001, *I*^2^ = 53.6%, 6 comparisons); and −0.44 on the domain of social support (*P*=0.020, *I*^2^ = 57.9%, 4 comparisons). The pooled effect on other domains ranged from −0.44 to 0.16 and did not reach statistical significance (Figure 3).

### 3.5. Subgroup Analysis

**Figure 4:**
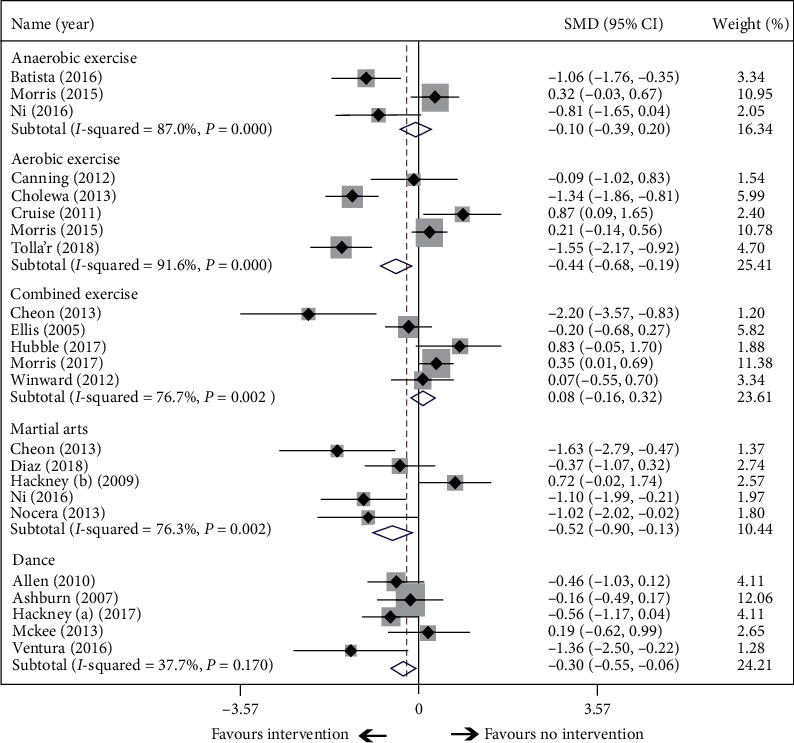
Subgroup analysis according to exercise types.

Exercise interventions were classified into five classes: anaerobic exercise, aerobic exercise, martial arts, dance, and the combined exercise of anaerobic and aerobic training (Table 1). The effect was significant for aerobic exercise (SMD = −0.44, 95% CI = −0.68 to −0.19, *P* < 0.001, 5 comparisons, Figure 4), martial arts (SMD = −0.52, 95% CI = −0.90 to −0.13, *P*=0.008, 5 comparisons), and dance (SMD = −0.24, 95% CI = −0.55 to −0.06, *P*=0.014, 5 comparisons), but it was nonsignificant for anaerobic exercise (*P*=0.509) and combined exercise (*P*=0.505).

**Figure 5:**
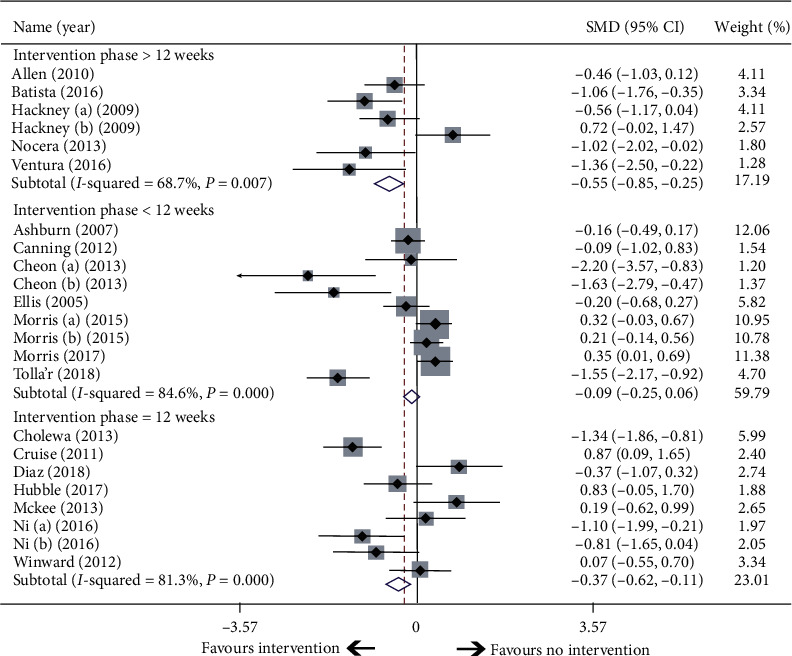
Subgroup analysis according to intervention period.

The mean intervention period was 12 weeks. Trials were divided into three categories according to the intervention period: longer than 12 weeks, less than 12 weeks, and exactly 12 weeks. The results showed that intervention lasting 12 weeks or longer resulted in statistically significant improvements in QoL with effects of −0.37 and −0.55, respectively. The longer intervention duration was associated with greater benefit (Figure 5).

The included studies were also classified into four subgroups according to the different types of QoL scales used. In the subgroup where QoL was evaluated by PDQ-39, a significant improvement in QoL was found between the exercise and control groups (*P*=0.005, 19 comparisons, Supplementary Appendix 3). The other three QoL scales employed were PDQL, EQ-5D, and SIP-68, but sample sizes were too small to gain meaningful results.

### 3.6. Meta-Regression

A meta-regression analysis across trials revealed significant predictors of the pooled effect, including the country in which the trial was performed (adjusted *R*^2^ = 59.0%; *P*=0.011), the region (adjusted *R*^2^ = 47.3%; *P*=0.0119), and the percentage of male participants (adjusted *R*^2^ = 24.3%; *P*=0.011). Publication year, mean age of participants, exercise type, intervention duration, and scale types were not significant predictors with *P* > 0.05. A multivariable meta-regression analysis found that the two factors of country and gender composition could explain 78.6% of between-study heterogeneity when combined (*P*=0.002).

### 3.7. Further Investigation of Heterogeneity

There was high heterogeneity among included studies (*Q* = 115.10, d*f* = 22, *P* < 0.001, *I*^2^ = 80.9%). Subgroup analyses and meta-regression implied that the heterogeneity was partially due to the country and gender composition of participants. The sensitivity analysis showed that none of the studies influenced the results significantly (Supplementary Appendix 4). A funnel plot was constructed to assess publication bias (Supplementary Appendix 5), with slight asymmetry found by Egger's test (*P*=0.027) and Begg's test (*P*=0.039). A trim and fill analysis was also conducted in order to identify and correct for funnel plot asymmetry. However, this method did not identify asymmetry and as such data remained unchanged.

## 4. Discussion

This review provides evidence that exercise interventions could significantly improve QoL in PD patients. The improvement was most apparent in the domain of mobility, ADL, and social support. Subgroup analyses revealed that aerobic exercise, dance, and martial arts provided significant benefits in QoL for PD patients. Anaerobic exercise and combined intervention, however, showed no benefits in QoL. Furthermore, longer duration of exercise intervention was related to greater improvement in QoL.

Previous meta-analyses have yielded inconsistent results regarding the impact of exercise on the QoL of PD patients. For example, Tomlinson et al. synthesized the data of PDQ-39 from seven trials, but did not find a significant benefit of physiotherapy intervention on QoL [4]. Our meta-analysis included more recent evidence, including a total of 1,143 participants from 20 studies, and our findings support the benefits of exercise for QoL in PD patients.

Besides the overall benefit, we also found that different types of exercise impacted QoL differently. Aerobic exercise, dance, and martial arts provided significant improvements in QoL, whereas anaerobic exercise did not lead to substantially different QoL relative to the control group. Previous studies revealed that, while resistance training can improve the muscle strength and reduce bradykinesia in PD patients, it may be tedious and lacking in motor complexity, and as such does not appear to contribute to QoL improvement [25, 28, 34]. In contrast to resistance training, dance can provide diverse stimulation, social, and emotional interaction, and the physical activity with a variety of brain areas activated [35, 36], which may contribute to better subjective feelings and result in an improvement in QoL. Two reviews of the effects of dance on QoL in PD reinforce these positive findings [37, 38]. More research is needed to investigate the physiological and clinical effects of different exercises, however.

Among the eight domains of the PDQ-39, only mobility, ADL, and social support were significantly improved after exercise. Previous research has suggested that exercise-based interventions, such as trunk exercises, aquatic exercise, and Tai chi may be effective for improving mobility, functional capacity, balance, and gait in PD patients [39–41]. This partially accounts for the improvements in mobility and ADL domains in our analysis. But the results in other domains were inconsistent with previous studies. Previous studies have provided evidence that exercise confers the additional benefit of a reduction in depressive symptoms and cognitive impairment [42, 43]. Additionally, studies of rat models of PD showed that specific exercise ameliorated cognition deficits and depressive symptoms and upregulated the expression of tryptophan hydroxylase and serotonin 1A, providing more evidence for the effectiveness of the physical activity on nonmotor aspects of PD [44]. In this review, however, there were only four studies included in the domain analysis [22, 27–29], and sample sizes were insufficient to gain convincing results.

The mean intervention period of studies included in our meta-analysis was 12 weeks. At intervention durations of 12 weeks or longer, the benefit of exercise in QoL became statistically significant. This cut-off value of intervention duration was consistent with that found by Mak et al. Strength training, aerobic training, Tai chi, or dance sustained for at least 12 weeks was associated with a long-term benefit on mobility [45]. Among the included comparisons in this review, the outcomes were assessed shortly after intervention had ceased, and the longest intervention duration was six months. Studies based on longer phases of intervention need to be conducted.

## 5. Limitations

There are several limitations of this systematic review and meta-analysis. First, there is a paucity of studies reporting detailed information of QoL domains. Second, none of the intervention periods lasted more than six months. Third, the methodological quality of the included trials was variable. All studies were at high risk of performance bias, as it was not possible to blind participants to group allocation. Fourth, there was high heterogeneity in the meta-analysis. However, we investigated heterogeneity and utilized a random effects model in our analysis.

## 6. Conclusions

This review suggests that exercise interventions, especially aerobic exercise, dance, and Tai chi significantly improve overall QoL of PD patients. This benefit was significant when the period of intervention was at least 12 weeks. Further research is needed to investigate the impact of exercise on different QoL domains, as well as the impact of longer durations of intervention.

## Figures and Tables

**Table 1:** Characteristics of studies included in the meta-analysis.

First author (year)		Population characteristics						Intervention					Assessment	
		Country	*N*, intervention/control	Mean age (years)	% Male	Mean duration of disease (years)	Mean UPDRS III score	Exercise type	Intervention	Length of each session (minutes)	Frequency (times/week)	Duration (weeks)	Assessment points (weeks)	QoL measure
Allen (2010) [6]		Australia	48, 24/24	67.0	54.2	8.0	29.5	Combined exercise	Strengthening and balance exercises	40–60	3	25	25	PDQ-39
Ashburn (2007) [16]		UK	142, 70/72	72.1	60.6	NA	21.0	Combined exercise	Exercise menu composed of muscle strengthening, range of movement, balance training, and walking	60	1	6	8	EQ-5D
Silva-Batista (2016) [30]		Brazil	39, 26/13	64.2	74.4	NA	44.1	Anaerobic exercise	Resistance training	60	2	12	12	PDQ-39
Canning (2012) [17]		Australia	20, 10/10	61.8	50.0	5.7	19.4	Aerobic exercise	Treadmill walking	30–40	4	6	6	PDQ-39
Cheon (2013) [18]		Korea	23, 7/9/7	64.4	0.0	5.6	28.0	Type A: combined exercise and type B: martial arts	Type A: combined stretching-strengthening exercise and type B: Tai chi	50–65	3	8	8	PDQL
Cholewa (2013) [19]		Poland	70, 40/30	70.2	65.7	7.7	21.8	Aerobic exercise	Rehabilitation exercises	60	2	12	12	PDQ-39
Cruise (2011) [20]		Australia	34, 17/17	60.0	52.9	5.7	NA	Aerobic exercise	Progressive anabolic and aerobic exercise	60	2	12	12	PDQ-39
Vergara-Diaz (2018) [7]		USA	32, 16/16	63.9	50.0	2.9	23.5	Martial arts	Tai chi	60	3	25	12	PDQ-39
Ellis (2005) [21]		USA	68, 35/33	63.5	75.0	NA	30.2	Combined exercise	Rehabilitation program including stretching exercises, strengthening exercises, functional training, gait training, balance training, and so on	90	2	6	6	SIP-68
Hackney (2009) [22]		USA	75, 38/17/20	66.4	81.8	7.6	27.1	Type A: dance and type B: martial arts	Type A: dance (Waltz and Foxtrot + Tango) and type B: Tai chi	60	2	13	13	PDQ-39
Hubble(2017) [23]		Australia	22, 11/11	65.4	68.2	6.8	19.4	Combined exercise	Falls prevention exercise	90	1	12	12	PDQ-39
McKee (2013) [24]		USA	33, 24/9	70.0	60.6	7.1	27.9	Dance	Tango	90	2	12	12	PDQ-39
Morris (2015) [25]		Australia	210, 70/69/71	67.9	66.7	6.5	11.6	Type A: anaerobic exercise and type B: aerobic exercise	Type A : progressive resistance strength training and type B : movement strategy training	120	2	8	8	PDQ-39
Morris (2017) [26]		Australia	133, 67/66	71.0	60.2	NA	35.5	Combined exercise	Home program comprising progressive resistance strength training, movement strategy training, and falls education	60	2	6	6	PDQ-39
Ni (2016) [27]		USA	27, 15/12	72.8	63.0	6.5	NA	Martial arts	Power yoga program	60	2	12	12	PDQ-39
Ni (2016) [28]		USA	26, 14/12	73.0	54.2	6.3	30.7	Anaerobic exercise	High-speed resistance training combined balance and agility drills	NA	2	12	12	
Nocera (2013) [29]		USA	23, 17/6	65.9	52.4	7.7	NA	Martial arts	Tai chi	60	3	16	16	PDQ-39
Tollar (2018) [31]		Netherlands	64, 35/29	67.4	52.7	NA	NA	Aerobic exercise	A high-intensity and high-frequency sensorimotor and visuomotor agility training program	60	5	3	3	PDQ-39
Ventura (2016) [32]		USA	15, 8/7	71.1	13.3	NA	NA	Dance	Dance	75	NA	19	19	PDQ-39
Winward (2012) [33]		UK	39, 20/19	64.1	59.3	NA	NA	Combined exercise	Aerobic sessions and strength sessions	30	7	12	12	PDQ-39

EQ-5D, EuroQol; NA, not available; PDQ-39, 39-item Parkinson's disease questionnaire; PDQL, Parkinson's disease quality of life questionnaire; QoL, quality of life; and SIP-68, sickness impact profile.

## Data Availability

The data supporting this meta-analysis are from previously reported studies, which have been cited. The processed data are available from the corresponding author upon request.

## Supplementary Materials

Supplementary Materials Appendix 1: search strategy. Appendix 2. (A) Risk of the bias graph: review authors' judgments about each risk of the bias item, presented as percentage across all included studies. (B) Risk of bias summary: review authors' judgments about each risk of the bias item for each included study. Appendix 3. The subgroup analysis according to scales for assessing life quality. Appendix 4. Sensitivity analysis. Appendix 5. Funnel plot.
